# Dimensional Transformation of Percolation Structure in Mixed-Matrix Membranes (MMMs)

**DOI:** 10.3390/membranes13090798

**Published:** 2023-09-16

**Authors:** Alexey Grekhov, Yury Eremin

**Affiliations:** Molecular Physics Department, Moscow Engineering Physics Institute, National Research Nuclear University, 115409 Moscow, Russia

**Keywords:** percolation effects, mixed-matrix membrane, anisotropy, tubular particles

## Abstract

A large number of studies of mixed-matrix membranes (MMMs) have confirmed the possibility of obtaining new materials with unique transport properties, including for solving specific problems in the separation of mixtures of liquids and gases. The choice of particles with a given affinity for the matrix and separable components allows researchers to adjust the selective properties of MMMs in a wide range, which changes the properties of MMMs in a wide range. However, even within the framework of the most complex percolation mechanism of the formation of the MMM structure, it is possible to explain only some of the observed effects. In particular, questions about the required particle concentration and fluctuation of properties in various MMM samples are still the subject of research. The results of the numerical modeling of such structures presented in this paper determined the possible causes of the observed deviations of the experimental results, for example, particle size dispersion, agglomeration, and interaction with the matrix. According to our research, the key factor that qualitatively changes the parameters of percolation structures is the ratio of the geometric dimensions of the system. We have confirmed in a wide range a significant change in the conditions of cluster formation and its power at different particle diameters and lengths (traditional parameters in percolation studies). But in our work, we additionally studied the effect on the cluster parameters of the interfacial layer and the anisotropy of the matrix (the transition from the cube to the film). The results obtained show that changing the parameters of the matrix–particle interaction affects agglomeration, and the degradation of the percolation structure is possible. That is, with an increase in concentration, the parameters of the percolation cluster, its power, and the probability of formation, may decrease. But even more negative changes in percolation structures are observed during the transition from a volumetric matrix to films. The anisotropy of space leads to the formation of percolation through the film in certain areas at low concentrations of particles. At the same time, in a significant part of the matrix, percolation between the film surfaces will be absent, and the effect of changing the properties of MMMs in the matrix as a whole decreases. Our study explains the observed instability of MMM properties at fixed concentrations and parameters of embedded particles, including the effect of reducing the influence of particles with increasing concentration.

## 1. Introduction

One of the actual directions in creating materials with new functional properties is the introduction of nanometer-sized particles with various geometric parameters and physical properties into polymer materials. Most often, various ceramic and carbon-containing particles are used for implementation, which allows researchers to vary the properties of the selected matrix in a wide range [1,2]. It is known that the transport and selective characteristics of MMMs vary in a wide range depending on the type of polymer and particles [3,4,5,6,7,8]. In such structures, the properties of MMMs can change according to different scenarios and there is still no universal model that describes the observed effects. It is known that the characteristics of MMMs depend not only on the type of embedded particles, but also on the parameters of the structures formed by the particles in the matrix [9,10,11,12,13]. 

Experiments show that the properties of MMMs change in a threshold manner when a certain concentration of embedded particles is reached. Such a sharp change in properties can be explained only within the framework of the percolation approach, assuming that at a critical concentration a connected structure (percolation cluster) is formed, which determines the additional flow of the target component in the MMMs. According to the classical percolation theory, when the critical concentration is exceeded, percolation structures become homogeneous and a monotonous change in the properties of such materials should be observed. However, as experimental studies show, fluctuations in properties are observed in real MMMs and the results obtained may differ significantly from forecasts. At the same time, the same material may show an improvement in properties for some gases and a deterioration for others. Despite the twenty-year history of research on this problem, these effects are regularly observed at present.

In work [14], in PVA/PEG membranes filled with multilayer carbon nanotubes with a modified surface (SE-MWCNTs), with a CNT concentration of 0.5%, a maximum permeability to CO_2_ and a selectivity of CO_2_/N_2_ and CO_2_/CH_4_ are observed. The permeability to other gases first increased and then decreased, i.e., it varied non-linearly in a narrow range.

When adding functionalized poly(ethyleneglycol) (PEG) carbon nanotubes, it was found that the permeability to CO_2_ first increases and then decreases with an increase in the content of CNT-PEG in the dry state [15]. Water vapor in the gas stream further improves the permeability to CO_2_. At a relative humidity of 100% for a hybrid membrane containing 3 wt.% CNT-PEG, a permeability to CO_2_ of 369.1 barrels was obtained with a CO_2_/N_2_ selectivity of 110.8, which exceeds the upper limit of Robson. At the same time, the permeability of CNT-PEG to CO_2_ varies non-linearly depending on the concentration of CNTs. An increase in the mass concentration of CNTs from 0 to 3% leads to an increase in the permeability to CO_2_ from 90 to 260 barrels, and an increase in the concentration of CNTs from 3 to 20% leads to a decrease in the permeability to CO_2_ from 260 to 110 barrels.

Carbon nanotubes were wrapped by poly(styrenesulfonate) (PSSA) and poly(vinylpyrrolidone) (PVP). Wrapped nanotubes were incorporated into poly (vinyl alcohol) matrix [16]. Depending on the MMM manufacturing method, two different effects were obtained from the introduction of CNTs. In the first case, an increase in the concentration of CNTs from 0 to 3% within the margin of error did not affect the flow of the azeotropic IPA/H_2_O feed mixture at 50 °C, and an increase in the concentration from 3% to 5% led to a doubling of the flow. In the second, an increase in the CNT concentration from 0 to 3% led to a 3.5-fold increase in the flux, and a further increase in the flux led to a decrease in the flux from 0.14 to 0.08 kg/(m^2^∙h).

The incompatibility between filler and polymer chains accompanied by particle agglomeration has a detrimental effect on the performance of mixed-matrix membranes (MMMs) [17]. To obviate intermolecular forces of different additives, functional groups (-COOH, -NCO, and -NH_2_) were grafted on the surface of multi-walled carbon nanotubes (MWCNTs) which were then incorporated as fillers in the poly(ether-block-amide) (PEBA) polymeric matrix in the range of 0.1–1 wt% loading. The permeability of MMMs to all the studied gases first increased with an increase in the concentration of CNTs, and then decreased.

In work [18], MMMs were fabricated from a highly microporous Tröger’s base (TB) ladder polymer matrix (ITTB) containing triptycene as contortion center, and COOH-functionalized single-wall carbon nanotubes (CNTs) as filler ranging from 0.6 to 2.0 wt%. By the strong acid–base interaction between COOH (from the CNTs) and tertiary-amine (from the TB) as well as π-π interaction between the triptycene unit and the CNT skeleton, the resulting MMMs had good compatibility. The permeability of the obtained MMMs to all gases (O_2_, H_2_, CO_2_) varied non-linearly with the CNT concentration. An increase in the concentration of CNTs from 0 to 1% led to an increase in the permeability to all gases under study, and a further increase in the concentration from 1 to 2% led to a decrease in the permeability to all gases except hydrogen; for hydrogen, the permeability did not change within the error. In recent studies on the introduction of CNTs into polymers, it is reported that the upper limit of Robson has been overcome [14,15,16,17,18].

In two main approaches, such materials are described within the framework of self-consistent models (for example, Maxwell, Kang–Jones–Nair, etc. [19]) in which the interaction of particles is not taken into account. However, these models can only explain monotonous changes in the properties of MMMs. Some of the observed threshold effects are described within the framework of the percolation theory approach (for example, the models of Shen, Kirkpatrick, etc. [20]), which allows us to take into account cooperative effects in such structures. This approach is applicable to both convective and diffusive transport in MMMs and allows us to take into account the influence of morphology and the interaction of particles and the matrix.

In work [21], the permeation process of gas molecules through MMMs embedding impermeable spherical and cuboid nanoparticles of different aspect ratios was simulated using a three-dimensional finite-difference solution of Fick’s second law of diffusion. The authors showed that, for the same volume fraction, the relative permeability of MMMs with cuboid nanoparticles with an aspect ratio larger than unity was lower than the relative permeability of membranes with spherical nanoparticles. This led to an analytical model, which involves only two geometrical parameters, the ratio of the projected area available for diffusion and the maximum surface area for diffusion, which depends on the aspect ratio of the nanoparticle, and the relative thickness of the nanoparticle. A model for the prediction of the mass transport through ideal MMMs for pervaporation and gas separation processes has been introduced [22]. A resistance-based model was used in conjunction with a three-dimensional finite difference (FD) numerical solution to derive a semi-empirical model for calculating the effective permeability of ideal mixed-matrix membranes. The authors claim that the extended RB model was theoretically able to accurately predict the effective permeability of ideal MMMs over a large range of filler volume fraction. However, in this work, the authors also consider the ideal morphology, which is difficult to achieve in polymers with embedded nanoparticles due to the physical and chemical characteristics of the nanoparticles–matrix interface [23]. However, these models and parameters of percolation structures are proposed for systems in which the dimensions of the matrix exceed the dimensions of embedded particles, which is not true for real selective layers. This fundamental difference, in our opinion, determines all the observed deviations of model predictions from experimental results. As will be shown below, the surface effects (the proportion of the modified material) and the commensurability of the matrix and particle sizes critically affect the conditions for the formation of the percolation structure.

**Figure 1:**
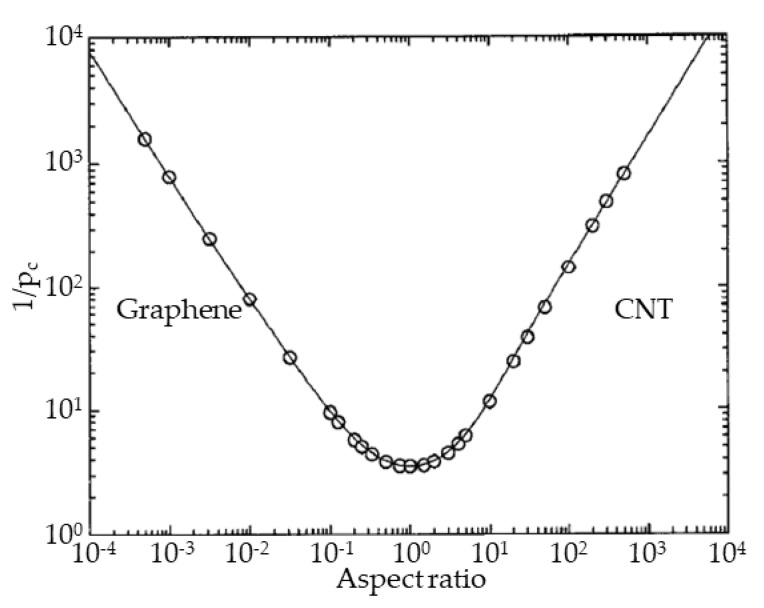
Percolation concentration for objects with different aspect ratio.

The modeling of percolation structures in isotropic 2D and 3D systems has shown that an increase in the aspect number of particles (transition to tubular or planar objects) leads to a significant decrease in percolation concentration (Figure 1) [24]. A non-zero probability of physical interaction (contact) with embedded 3D particles exists in an effective volume near the particle, which can be described by a sphere with a diameter equal to the largest particle size. However, the proportion of space that this object actually occupies in the matrix will be determined by all three of its dimensions. Therefore, with an increase in the aspect number of particles (the ratio of two characteristic linear ones), the fraction of the particle volume in the effective volume decreases. At the same time, the probability of formation of a percolation cluster (interaction of individual particles) increases with an increase in the effective volume, or, accordingly, with an increase in the aspect number. The extremum of the critical concentration observed in Figure 1 corresponds to particles with an aspect number close to one, at which the physical and effective volume are equivalent (without taking into account the effects of particle interaction through the matrix), and corresponds to the theoretical limit values of the percolation threshold in 3D isotropic space. At the same time, both for tubular particles with a large aspect number (CNTs) and for flat particles with a small aspect number (graphene), the effective volume is significantly larger than the real one and the percolation transition is observed at lower concentrations. Experimentally, this effect was confirmed for various MMMs with CNTs and other particles [3,9,10,11,25,26]. However, in other studies, there is a significant deviation of properties similar to MMMs [27,28,29,30,31,32,33,34,35,36]. It is precisely this difference in properties for identical materials that confirms our thesis about fluctuations in the parameters of the percolation transition, which cannot be explained only by particle sizes.

We expect that, in addition to the properties of the particles, the percolation parameters are controlled by the geometry of the membranes. Complex experimental and model studies of the conditions for the formation of a percolation cluster and its characteristics make it possible to build a consistent model of such structures in MMMs, which is necessary for the further development of the technology of synthesis and production of MMMs.

## 2. Calculation

The threshold change in the properties of materials with embedded nanoparticles is considered in the theory of percolation, in which it is believed that an abrupt change in the properties of materials with embedded nanoparticles occurs as a result of the formation of interconnected new structures, which are called a percolation cluster. This approach is often used for descriptions of changes in the electrical and thermal conductivity of polymers when nanoparticles are introduced into them.

Mass transfer in a polymer with carbon nanotubes embedded in its structure is possible:

(1) Through the polymer;
(2) Through the polymer/CNT interfacial layer, which results from poor adhesion between polymer chains and CNTs;
(3) Through the internal cylindrical channels of the CNTs.

And if we consider the flow through regions (1)–(3) as independent, then we can write the rule of mixtures, in which the fraction of the volume of the membrane with high permeability should be related to the volume of the percolation cluster, as

(1) $$K_{\mathrm{MMM}}=K_{x}\cdot C_{x}\left(R,L,\mathrm{dR}\right)+K_{p}\cdot \left(1-C_{x}\left(R,L,\mathrm{dR}\right)\right)$$

where C_x_ is the fraction of the polymer volume occupied by the percolation cluster, K_x_ is the permeability coefficient of the percolation cluster, K_p_ is the polymer permeability coefficient, and dR = R – R_0_ is the thickness of the interfacial layer. The thickness of polymeric membranes with embedded CNTs differs slightly from the length of the CNTs; such a system cannot be considered infinitely large when calculating the parameters of a CNT percolation cluster, and surface effects must be taken into account. Expression 1 has a general form for a composite material parameter and illustrates the linear correlation of properties in a two-component material based on the rule of mixtures. It shows that the contribution of each component is proportional to its concentration and the observed nonlinear changes in properties can be associated with a nonlinear change in the number of particles affecting the properties of the matrix.

To determine the parameters of a percolation cluster of CNTs in systems of finite size, a software package in C# was developed, which allows researchers to determine the probability of formation and the strength of a percolation cluster for various geometric objects (sphere, sphere with an impenetrable core, spherical cylinder, spherical cylinder with an impenetrable core, segment, circle, and rectangle), and set the distribution function of geometric objects by size.

Due to the fact that carbon nanotubes are the object of study in this work, the algorithm for calculating the parameters of a percolation cluster was considered using the example of spherocylinders with impenetrable cores in the form of a spherocylinder.

In our model, each spherical cylinder is surrounded by a shell with a thickness of dR = R − R_0_, which forms regions of the polymer, the permeability of which increases due to the interaction with the surface of the nanotubes, and is determined by the interaction between the CNTs and the polymer. Spherocylinders form a cluster when shells overlap, and if a cluster connects opposite faces of a 3D matrix (upper and lower), then it is considered percolative. To achieve this, a three-dimensional matrix is randomly filled with spherical cylinders, the distance between them is determined, a list of formed clusters is formed, and it is checked whether there is a percolative one among them.

To determine the probability of formation of a percolation cluster, a given number of iterations is carried out with the same number of spherocylinders added to the system.

To determine the strength of the percolation cluster, the ratio of the number of spherical cylinders that make up the percolation cluster to the number of spherical cylinders added to the system is calculated as

(2) P∞=N∞N

where N_∞_ is the number of spherical cylinders that make up the percolation cluster, and N is the number of spherocylinders added to the system.

The volume concentration of spherocylinders was determined as

(3) $$C_{x}=\left(\frac{4\cdot \pi }{3}\cdot R_{0}^{3}+\pi \cdot R_{0}^{2}\cdot L\right)\cdot N/V$$

where R_0_ is the radius and L is the length of the spherocylinders, N is the number of spherocylinders, and V is the volume of the matrix. 

The average value of the volume fraction occupied by high-permeability regions of the polymer is

(4) $$C_{x}=\left(\frac{4\cdot \pi }{3}\cdot (R^{3}-R_{0}^{3})+\pi \cdot (R^{2}-R_{0}^{2})\cdot L\right)\cdot \frac{N_{x}}{V}$$

Here, $N_{x}$ is the number of spherocylinders in the percolation cluster. To verify the algorithm and program, the percolation threshold was calculated for various geometric objects [37]. 

Since a spherical cylinder with an impenetrable core in the form of a spherical cylinder is a CNT model in our work, we will use CNT from now on in the text. We will give the dimensionless value of the geometric dimensions of the matrix and spherocylinders with units of measurement tied to the dimensions of polymer membranes and CNTs.

## 3. Results and Discussion

### 3.1. Influence of CNT Length

**Figure 2:**
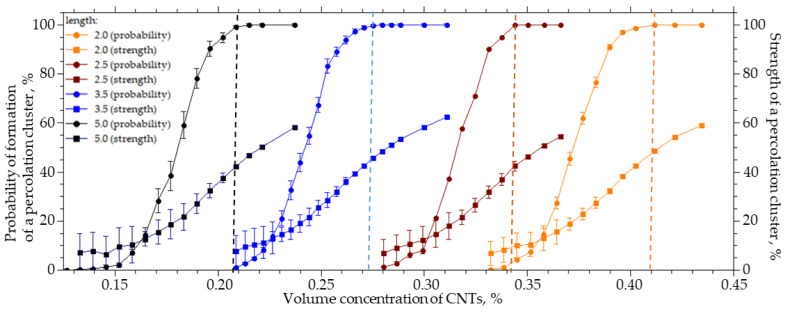
The probability of the formation and strength of a percolation cluster from the volume concentration of CNTs with a length from 2 to 5 µm.

For a system sized 25 × 25 × 25 µm (width X, height Y, length Z), the length of the CNTs varied from 2 to 5 µm, the radius R_0_ was equal to 25 nm, and the outer radius R was equal to 125 nm. Figure 2 shows the results of the numerical simulation. 

An increase in the CNTs’ length from 2 to 5 µm leads to a decrease in the CNTs’ concentration, at which the probability of the formation of a percolation cluster is 100%, from 0.41 to 0.20%. In this case, the cluster strength does not exceed 50%, and a further increase in the CNT concentration leads to an increase in the cluster strength (Figure 2). It can be seen that a decrease in the length of CNTs leads to an increase in the concentration of the CNTs which must be introduced in order to form a percolation cluster. That is, the contribution of the percolation cluster is halved. The length of the CNTs has a significant effect on the percolation concentration and the structure of the percolation cluster. A decrease in its strength leads to the formation of a looser structure and reduces the contribution to the transport properties of the MMMs from the embedded phase, despite the fulfillment of percolation conditions.

It is important to note that the transition from a system without a percolation cluster to a system in which a percolation cluster is formed with a probability of 100% occurs with a slight change in concentration. For example, for 5 µm long CNTs, it is necessary to increase the CNT concentration from 0.12% to 0.21%, and for 2 µm long CNTs, it is necessary to increase the concentration from 0.32% to 0.40%. The strength of the percolation cluster shows its branching and the proportion of the inserted CNTs that participates in its formation. For example, in order to increase the strength from 10% to 80%, it is necessary to increase the volume concentration of CNTs with a length of 5 µm from 0.18% to 0.33% (1.8 times).

**Figure 3:**
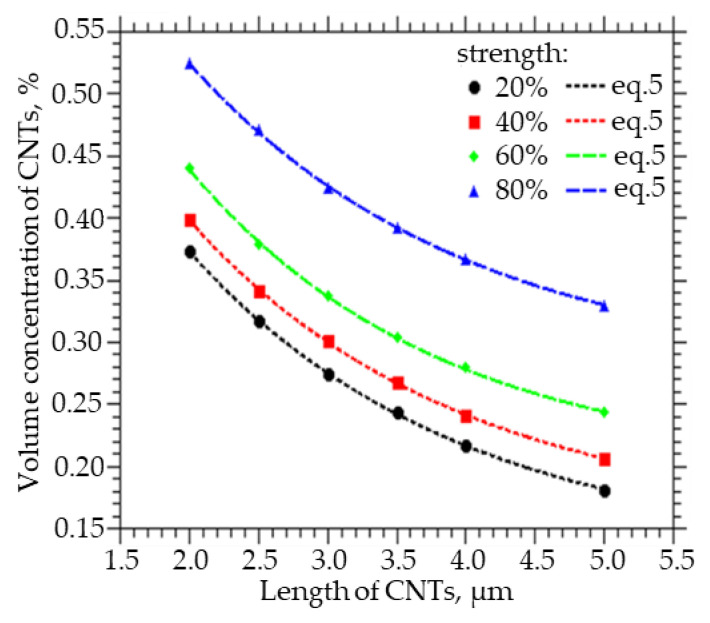
The volume fraction of CNTs that must be embedded in order to obtain the strength of the percolation cluster equal to 20, 40, 60, and 80% of the CNT length.

Figure 3 shows how the volume fraction of CNTs, which must be embedded in order to obtain a strength of the percolation cluster equal to 20, 40, 60, and 80%, varies with the CNT length. A decrease in the length of CNTs from 5 to 2 µm leads to an increase in the volume concentration of CNTs from 0.34 to 0.53% at a percolation cluster strength of 80%, from 0.25 to 0.45% at a percolation cluster strength of 60%, from 0.22 to 0.4% at a percolation cluster strength of 40%, and from 0.18 to 0.38% at a percolation cluster strength 20%. Also, from the approximation of the data, a function was obtained that can be used to calculate the concentration of CNTs of a given length, at which a percolation cluster with a strength of 20, 40, 60, and 80% will be observed:

(5) $$C\left(L\right)=y_{0}+A\cdot \exp\left(-L/t\right)$$

where y_0_ ranges from 0.1294 to 0.2742, A from 0.6809 to 0.6814, and t from 1.9458 to 1.9980.

### 3.2. Effect of CNT Interfacial Layer Thickness

**Figure 4:**
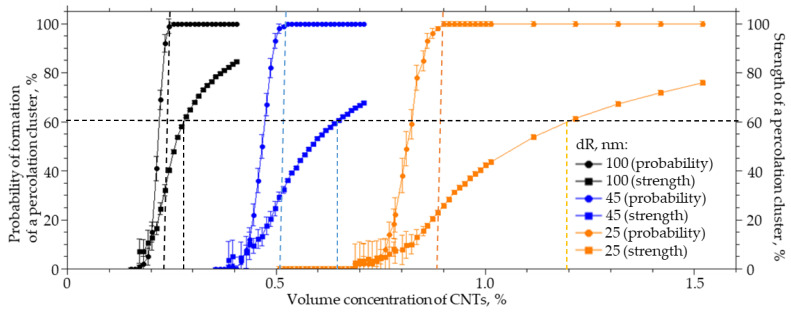
The probability of the formation and the strength of a percolation cluster from the volume concentration of CNTs with the thickness of the interfacial layer dR from 100 to 25 nm.

When introduced into polymers, CNTs are modified by grafting various functional groups, which leads to a change in the thickness of the interfacial layer. For a system sized 25 × 25 × 25 µm (width X, height Y, length Z), a numerical simulation of the effect of the thickness of the CNT interfacial layer on the probability of formation and strength of a percolation cluster was carried out. The thickness of the interfacial layer of CNT (dR) varied from 25 to 100 nm, the radius of the R_0_ was equal to 25 nm, and the length L was equal to 4 µm. An increase in the thickness of the interfacial layer from 25 to 100 nm leads to a nonlinear decrease in the CNT concentration at which the percolation cluster is formed with a probability of 100%. This change in the thickness of the interfacial layer leads to a decrease in the concentration of the CNTs which must be introduced into the polymer to form a percolation cluster with a probability of 100% and a strength of 60% by a factor of 4.3 (Figure 4).

The interaction of a polymer with a CNT surface strongly affects the concentration at which bonded CNT structures will be formed in the polymer. For different polymers and identically modified CNTs, or for one polymer and CNTs with different grafted functional groups, the concentration at which a percolation cluster will form can differ by several times. 

**Figure 5:**
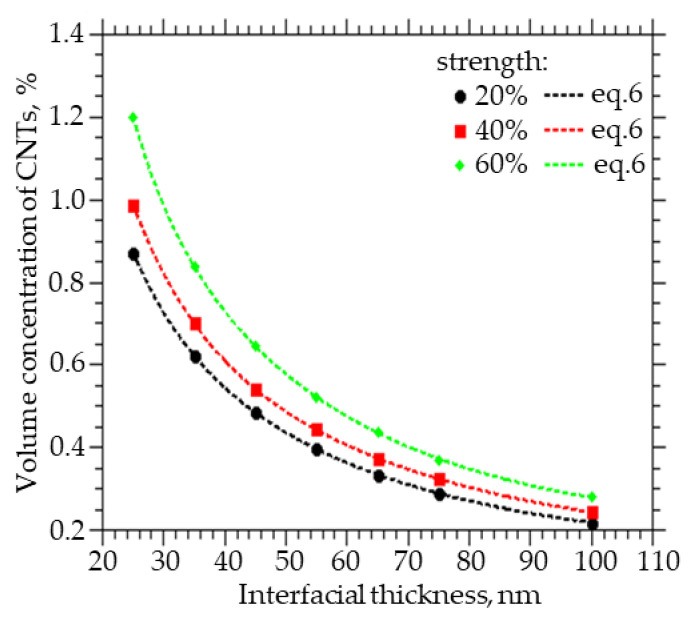
The volume fraction of CNTs that must be embedded in order to obtain the strength of the percolation cluster equal to 20, 40, and 60% of the thickness of the interfacial layer.

Figure 5 shows how the volume fraction of CNTs to be embedded varies with the thickness of the interfacial layer between the CNTs and the polymer in order to obtain a percolation cluster strength of 20%, 40%, and 60%. A decrease in the thickness of the interfacial layer between CNTs and polymer from 100 to 25 nm leads to an increase in the volume concentration of CNTs from 0.3% to 1.2% at a percolation cluster strength of 60%, from 0.25% to 0.98% at a percolation cluster strength of 40%, and from 0.22% to 0.4% at a percolation cluster strength of 20%.

Also, from the approximation of the data, a function was obtained that can be used to calculate the concentration of CNTs with a given layer thickness, which they change around themselves, and at which a percolation cluster with a strength of 20, 40, and 60% will be observed:

(6) $$C\left(x\right)=A_{1}\cdot \exp\left(-x/t_{1}\right)+A_{2}\cdot \exp\left(-x/t_{2}\right)+y_{0}$$

where x = R − R_0_, A_1_ ranges from 3.0383 to 1.7113, A_2_ from 1.0812 to 6.8893, t_1_ from 0.0088 to 0.0329, t_2_ from 0.0371 to 0.0071, and y_0_ from 0.1451 to 0.1973.

### 3.3. Effect of CNT Radius

**Figure 6:**
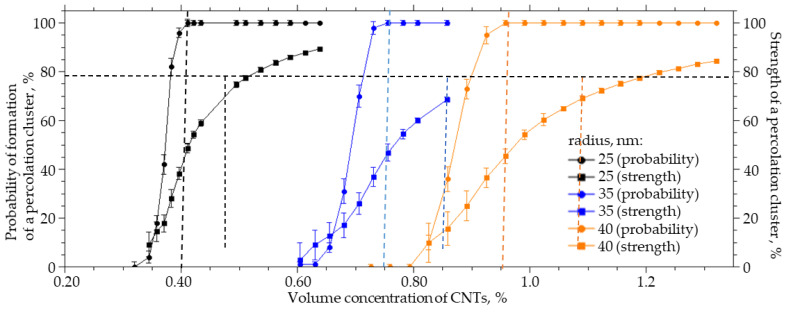
The probability of the formation and the strength of the percolation cluster from the volume concentration of CNTs with an inner radius of 25 to 40 nm.

CNTs can have different diameters. They can form bundle-shaped agglomerates as a result of interaction with side surfaces, which can lead to an increase in the radius of a spherical cylinder, which in this case is a model of a CNT agglomerate. For a system sized 25 × 25 × 25 µm (width X, height Y, length Z), a numerical simulation of the influence of the CNT radius on the probability of formation and strength of a percolation cluster was carried out. The radius varied from 25 to 40 nm, the thickness of the interfacial layer dR was equal to 100 nm, and the length L was equal to 2 µm. Figure 6 present the results of the numerical simulation. Increasing the radius of CNTs from 25 to 40 nm leads to a nonlinear increase in the concentration of CNTs that must be incorporated into the polymer to obtain a percolation cluster with a probability of 100% and a strength of 70% by a factor of 2.3. The interaction of carbon nanotubes with the side surface can lead to the formation of agglomerates in the form of “bundles”; then, the percolation cluster in the polymer will be formed not from individual CNTs but from their agglomerates. These agglomerates can be thought of as a spherocylinder with an increased radius. It follows from the simulation that this will lead to a decrease in the strength of the percolation cluster or its destruction.

**Figure 7:**
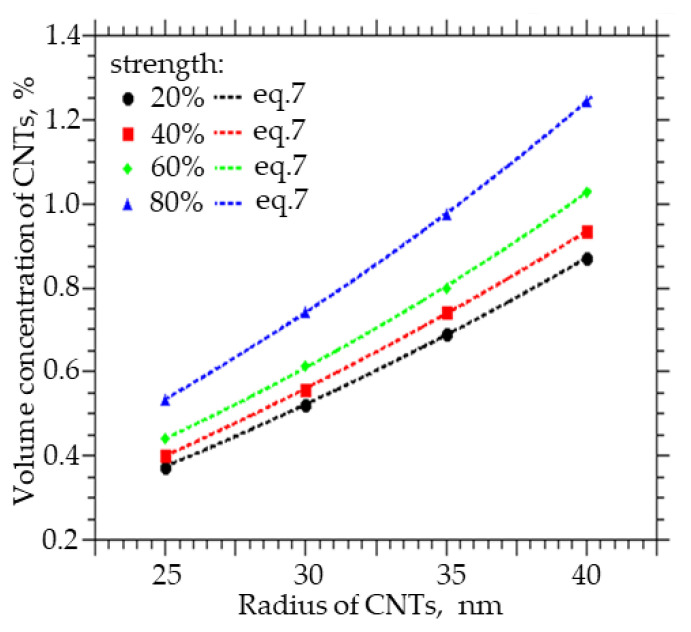
The volume fraction of CNTs that must be embedded in order to obtain the strength of the percolation cluster equal to 20, 40, 60, and 80% of the CNT radius.

Figure 7 shows how the volume concentration of CNTs, at which the specified value of the percolation cluster strength (20, 40, 60, and 80%) is reached, changes with the CNT radius R_0_. An increase in the radius from 25 to 40 nm leads to an increase in the volume concentration at which a percolation CNT cluster is formed in the polymer from 0.35 to 0.85% at a strength of 20%, from 0.4 to 0.84% at a strength of 40%, from 0.42 to 0.95% at a strength of 60%, and from 0.51 to 1.25% at a strength of 80%.

Also, from the approximation of the data, a function was obtained with the help of which it is possible to calculate the concentration of CNTs of a given diameter, at which a percolation cluster with a strength of 20, 40, 60, and 80% will be observed:

(7) $$C\left(R_{0}\right)=y_{0}+A\cdot \exp\left(R_{0}/t\right)$$

where A ranges from 0.7653 to 0.7902, t from 0.0932 to 0.0777, and y_0_ from −0.934 to −0.9693.

Modeling showed that the parameters of a percolation cluster are directly determined by the size of CNTs and the thickness of the interfacial layer, the size of which is determined by the interaction between the polymer and the CNTs. A model that will make it possible to predict the properties of polymeric membranes with embedded CNTs should take into account the geometric parameters of CNTs and the thickness of the interfacial layer.

### 3.4. Effect of Geometric Parameters of the System

If the size of the system becomes commensurate with the sizes of the objects from which the bound structures are formed, then surface effects begin to play an important role, and the parameters of the percolation cluster begin to depend on the ratio between the particle sizes and the film sizes. The thickness of the membranes and the length of the CNTs are comparable; therefore, the effect of the geometric dimensions of the system on the parameters of the percolation cluster was simulated. 

**Figure 8:**
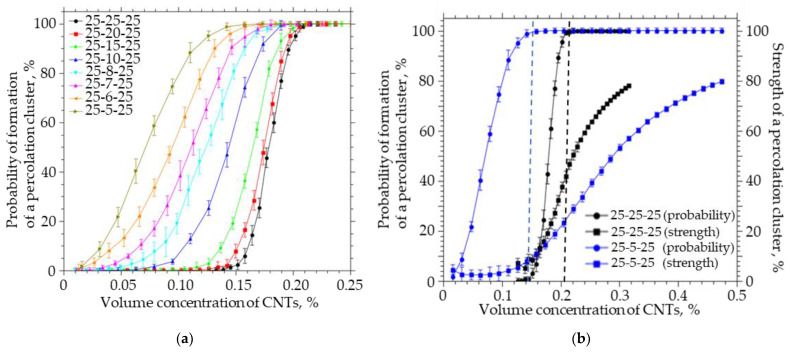
(**a**) The probability of the formation of a percolation cluster for anisotropic systems of different thicknesses (from 25 to 5). (**b**) The probability of the formation and the strength of the percolation cluster on the volume concentration of CNTs for anisotropic systems of different thicknesses (from 25 and 5).

A simulation was carried out in which the thickness of the system was reduced at a fixed length and width. The thickness of the system decreased from 25 to 5 µm, the width and length were fixed and equal to 25 nm, the CNT length was equal to 5 µm, the radius was 25 nm, and the thickness of the interfacial layer was 0.1 nm. The simulation results are shown in Figure 8.

Reducing the thickness of the system to the length of CNTs leads to a significant de-crease in the concentration of CNTs at which the percolation cluster begins to form, while the strength of such a cluster does not exceed 2%. To increase the strength of the percolation cluster in a system with a size of 25-5-25 to 80%, it is necessary to increase the concentration of CNTs by a factor of 31. It is important to note that the transition from an isotropic system to an anisotropic system with a thickness equal to the CNT length is accompanied by a decrease in the concentration at which a bond is formed between the upper and lower boundaries of the system from 0.14% to 0.01% (by a factor of 14). To achieve the probability of formation of a percolation cluster, it is necessary to increase the concentration of CNTs from 0.14% to 0.21% in the 25-25-25 isotropic system, and from 0.01 to 0.15 (15 times) in the 25-5-25 anisotropic system, while the percolation cluster strength will not be exceeding 5%. In order to achieve a percolation cluster strength of 80% in an anisotropic system, it is necessary to increase the concentration of CNTs to 0.47%.

It can be seen that an increase in the ratio of the length and width of the film to the thickness from 1 to 10 leads to a decrease in the strength of the percolation cluster from 48 to 2% (Figure 8b). Thus, in thin films, in which the thickness is comparable to the CNT length, the concentration at which a percolation cluster will form will be much lower, while the percolation cluster strength will not exceed several percentage points, i.e., it will be in a limited area and unevenly distributed over the polymer film.

## 4. Conclusions

The purpose of our study was to model the conditions for the formation of a percolation structure in MMMs and to study the “stability” of such a transition when the geometric characteristics of embedded particles change. Since the transport properties of the MMMs are associated with the formation of a percolation cluster, it becomes crucial to understand the real parameters of such a cluster in the MMMs—the concentration of formation, the cluster’s strength, and its stability—when changing the geometric characteristics of the structure. The relationship between the parameters of percolation structures and the properties of MMMs is not the subject of this study, since there are no representative comprehensive experimental studies of both functional and structural properties of MMMs. At the same time, as has been repeatedly shown, the characteristics of the percolation cluster and particle sizes affect various macroscopic properties of the MMMs and the prediction of the parameters of such structures allows researchers to vary the functional properties of the MMMs. Let us consider several scenarios in which an erroneous assessment of real geometry leads to the absence of the predicted properties of MMMs.

Strong affinity. Let us suppose that at a certain concentration of particles with an assumed radius and length (R_0_ = 25 nm, L = 4 µm) in a geometrically isotropic matrix, we obtained a percolation structure of a certain strength. As can be seen from Figure 4, with an assumed layer thickness of dR = 100 nm, we can expect the formation of a percolation cluster with a capacity of about 80% at a particle concentration of more than 0.35%. However, if the actual thickness of the modified layer is 65 nm, then the cluster strength at the same concentration will be about 20%. That is, the proportion of MMMs in which specific transport associated with embedded particles is implemented will decrease by four times, which will significantly worsen the transport properties of such MMMs.

Geometric anisotropy of the matrix. Aiming to obtain a good percolation, for certain particles (R_0_ = 25 nm, L = 5 µm, and dR = 100 nm) in a geometrically isotropic matrix, a CNT concentration of more than 0.2% was chosen, which guaranteed a 100% probability of forming a percolation cluster with a strength of about 50% (Figure 8). However, with a decrease in the actual thickness of the film, the cluster strength is reduced by more than two times already with the ratio of lateral and normal matrix sizes at 5 µm (Figure 8b). A further increase, despite the fulfillment of the concentration conditions of percolation (Figure 8b), may lead to zero strength of the percolation cluster, and in such systems there will be a strong heterogeneity of the transport properties of the MMMs.

Size of CNTs. Too high a concentration of CNTs with inaccurate determination of their characteristics can also lead to negative changes in the percolation structure. For example, an excessive amount (0.5%) of CNTs (R_0_ = 25 nm, L = 2 µm, and dR = 100 nm) has been added to the isotropic matrix. With such particle sizes, a percolation cluster is formed at concentrations of about 0.4% (Figure 6); however, an error in determining the actual radius of the particles (for example, R_0_ = 30 nm) leads to a situation where the concentration is insufficient to guarantee the formation of a percolation cluster, and the strength of such a percolation cluster is less than 20%. Thus, most of the formed MMMs will not contain a percolation structure. Errors in the actual CNT length lead to similar consequences. For example, for particles of a given size (R_0_ = 25 nm, dR = 100 nm, and length L = 5 µm), we determine the required concentration of 0.2%, with some excess. However, if the actual length of the CNTs is equal to 4 µm, the percolation cluster is practically not formed, since the probability is less than 10% (Figure 2) and its capacity is about 10%. In such a system, the change in transport properties will be significantly lower than expected and in a small number of experiments. Thus, an error in the geometric dimensions of the particles by 20–25% leads to an erroneous choice of the required concentration and, as a consequence, the absence of the necessary percolation structure in the MMMs.

**Figure 9:**
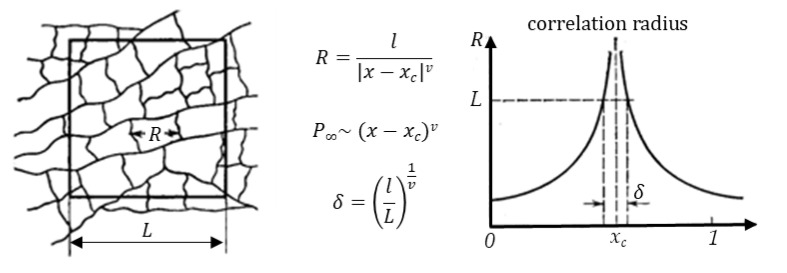
Correlation radius of percolation structures in the vicinity of percolation concentration for anisotropic systems.

A simultaneous implementation of the scenarios of variation and inaccuracy described above in the evaluation of geometric factors of MMM elements will lead to the instability of the observed properties of MMMs and the absence of percolation even at particle concentrations significantly exceeding the threshold value. From the point of view of the classical percolation theory, this result can be associated with the variance of probabilistic characteristics of percolation structures in systems of finite size. The transition of the system to a percolation state is possible both as a result of a change in the concentration of particles and by reducing the size of the system. For example, as shown in Figure 9, by cutting out cells (matrices) of various sizes from an infinite percolation cluster, we can fix the presence of a percolation cluster with a given frequency. At the same time, the characteristic scale of clusters and the strength of the resulting clusters depends both on the proximity to the critical concentration and on the size of the system.

In scaling theory, the cluster structure near the transition is related to the size of the problem space [38], and below the percolation threshold, the average cluster size determines the correlation length (Figure 9). At the percolation threshold, the correlation length diverges. Since the correlation length above the percolation threshold has a finite value, the infinite cluster is self-similar. One can consider the correlation length as the most probable distance up to which an infinite cluster is self-similar and can be considered as a fractal. At large length scales, the cluster structure is no longer self-similar, and the cluster can be considered as homogeneous. 

Thus, when describing the formation of percolation structures in films (systems of finite size), it is necessary to take into account multidirectional trends when changing the size and concentration of particles in the matrix. On the one hand, an increase in the concentration of particles and the correlation radius leads to an increase in the size of agglomerates, the formation of a percolation cluster, and the transition of the system to a homogeneous state. On the other hand, a decrease in the size of the matrix (system) leads to an increase in the variance of the probability of formation of a percolation cluster at a given concentration and the distribution of clusters by size.

Using the characteristics of the percolation cluster makes it possible to model the functional properties and parameters of the MMMs with a known geometry of all elements. At the same time, the influence of the percolation structures/geometric parameters on properties (for example, the permeability) depends on the characteristics of the process (phenomenon). For example, the permeability of the matrix and the structure of the percolation cluster determine the magnitude of the permeability of the MMMs and the area of observed changes. That is, nonlinear changes in various properties of MMMs are associated with the appearance of a percolation structure in the matrix when the contribution from individual particles becomes macroscopic (Expression 1). At the same time, the destruction of the cluster or a decrease in its capacity leads to a decrease in the effect of the cluster and the contribution of the matrix becomes decisive.

These processes lead to the formation of unstable conditions at certain points of the matrix and the percolation cluster becomes less “powerful”. That is, by reducing the thick-ness of the matrix, we can observe a deterioration in transport properties at a fixed concentration of particles, compared with the percolation cluster in thicker films. It is also possible to expect constant permeability with a change in concentration, in the case of a transition to a homogeneous region of particle concentrations. At the same time, at high concentrations of nanoparticles, a process of increasing the size of particle agglomerates can be observed, which leads to the degradation of the percolation structure.

## Data Availability

The data presented in this study are available on request from the corresponding author.
